# Pineal Gland Tumors: A Review

**DOI:** 10.3390/cancers13071547

**Published:** 2021-03-27

**Authors:** Gaia Favero, Francesca Bonomini, Rita Rezzani

**Affiliations:** 1 Anatomy and Physiopathology Division, Department of Clinical and Experimental Sciences, University of Brescia, 25123 Brescia, Italy; francesca.bonomini@unibs.it (F.B.); rita.rezzani@unibs.it (R.R.); 2 Interdipartimental University Center of Research “Adaption and Regeneration of Tissues and Organs (ARTO)”, University of Brescia, 25123 Brescia, Italy

**Keywords:** pineal gland, brain neoplasms, pineal germ cell tumors, pineal parenchymal tumor, pineal metastasis

## Abstract

**Simple Summary:**

Pineal neoplasms are tumors with different and variable morphological, histological, and radiological characteristics and, consequently different diagnosis and management. Due to their rarity, pineal tumors may be misdiagnosed. Pineal tumors, are divided into germ cell tumors, pineal parenchymal tumors and tumors that derive from adjacent structures. In this review, we report the clinical relevance of the main pineal gland tumors, underlining the importance of studying the triggering causes of pineal region carcinogenesis, to realize appropriate diagnosis and, consequently, better clinical management.

**Abstract:**

The pineal gland is a small, pinecone-shaped endocrine gland that participates in the biological rhythm regulation of vertebrates. The recognized major product of the pineal gland is melatonin—a multifunctional endogenous indoleamine. Accumulating evidence suggests that the pineal gland is important for preserving ideal health conditions in vertebrate. Tumors of the pineal region account for approximately 3–11% of pediatric brain neoplasms but fewer than 1% of brain neoplasms in adults. It is fundamental to expand advanced imaging techniques together with both clinical and laboratory knowledge, to help to differentiate among pineal neoplasms and thus facilitate accurate primary diagnoses and proper therapeutic interventions. In this review, we report the gross anatomy of the pineal gland and its functional significance and discuss the clinical relevance of pineal gland tumors, underlining the importance of identifying the leading causes of pineal region masses.

## 1. Introduction

### The Pineal Gland

**Figure 1:**
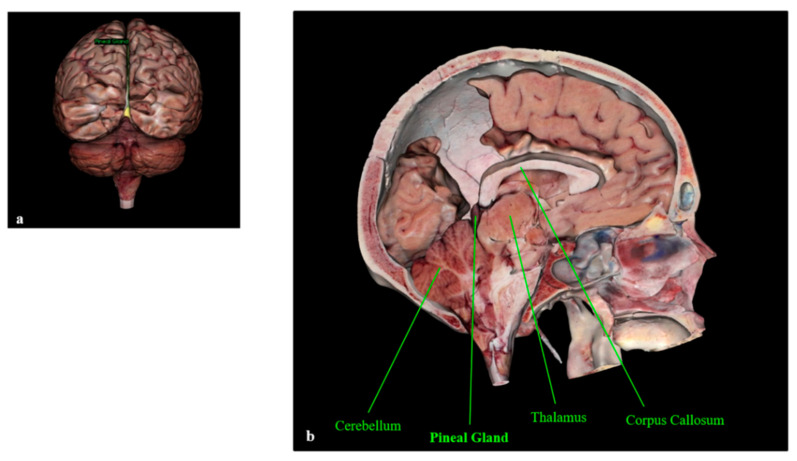
Human pineal gland (**a**) and its anatomic boundaries (**b**). The pineal gland is visible in yellow (**a**). Anatomage Inc.—Anatomage Table EDU. The 3D rendering of the cadaver data is from Anatomage Table.

The pineal gland is a pinecone-shaped neuroendocrine gland located in the epithalamus that participates in the biological rhythm regulation of vertebrates. The gland projects posteriorly and inferiorly into the quadrigeminal cistern, and the anatomic boundaries include the backside of the third ventricle wall forming the gland’s base, the splenium of the corpus callosum superiorly, and the thalamus surrounding both sides [1,2,3,4]. Figure 1 shows the location of the pineal gland in the human brain.

The pineal gland’s primary purpose is the production and immediate release of melatonin, a pleiotropic and multifunctional indoleamine [5,6,7,8,9,10], into the blood [11].

The pineal gland possesses two populations of cells: about 95% are pinealocytes with dendritic processes, and the other 5% are neuroglial supporting cells that resemble astrocytes. Both these types of cells can form neoplasms, as well as residual germ cells from primordial neural crest cell migration and cells derived from nearby structures [1,2,12,13,14,15,16]. The pineal gland may, unfortunately, harbor a variety of neurosurgical diseases such as pineal cysts, different pineal tumors, and vascular malformations, including cavernous, arteriovenous malformations and aneurysms.

## 2. Pineal Gland Tumors

**Figure 2:**
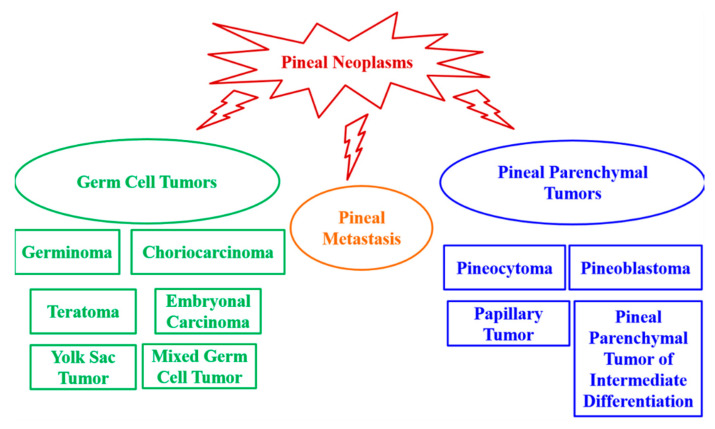
Pineal tumor classification.

Pineal neoplasms are fairly uncommon tumors, and they are predominantly childhood malignancies representing 3–11% of all pediatric brain tumors compared to <1% of brain tumors in adults [1,14,17,18,19,20]. Age, sex, and ethnicity may modulate the relative incidence of pineal neoplasms [21]. Pineal tumors are classified as: germ cell tumors, pineal parenchymal tumors and tumors that derive from adjacent anatomical structures. Germinoma is the most common pineal tumor, representing up to 50% of pineal tumors in Europe, the United States and Japan [1,22,23]. In a series of 370 pineal tumors in patients aged 3–73 years, it was observed that 27% were germinomas; 26% were astrocytomas; 12% were pineoblastomas; 12% were pineocytomas; 4.3% were ependymomas; 4.3% were teratomas; 2.7% were ganglioglioneuromas, lymphomas, meningiomas, metastases, and pineal cysts; 1.6% were mixed embryonal cell tumors (embryonal carcinomas)/malignant teratomas; 1.1% were choriocarcinomas; and 0.54% were oligodendrogliomas [24] (Figure 2).

Pineal region masses may produce nonspecific signs and symptoms, and they usually cause syndromes of mass effect, including headaches, aqueductal stenosis, and hydrocephalus, or compressive hypothalamic syndromes, such as diabetes insipidus and slowed growth [13,16,25]. A mass in the pineal area may also interfere with the normal function of the pineal gland. Pineal tumors associated with acute and rapidly progressive hydrocephalus may be clinically managed via external ventriculostomy, endoscopic third ventriculostomy, ventriculoperitoneal/ventriculoatrial shunts, or direct removal [26].

It is fundamental to expand advanced imaging techniques, together with both clinical and laboratory knowledge to help to differentiate among the pineal neoplasms and thus realize accurate primary diagnoses and correct treatment and patient management plans. Open surgical resection and stereotactic or endoscopic biopsy are needed for pineal tissue diagnosis [27,28]. However, stereotactic biopsy seems to be associated with a higher risk of hemorrhage in pineal region tumors [18,29]. In practice, the diagnosis of pineal region neoplasms is based on clinical presentation, imaging, and pathology results. Serum and cerebrospinal fluid (CSF) biomarkers complement these standard diagnostic techniques by providing additional data before invasive procedures are performed [1,12,30]. Therefore, research into novel diagnostic markers, therapeutic approaches and follow-up guidelines is fundamental.

In this review, we report the features and clinical relevance of the main pineal gland tumors, underlining the importance of studying the triggering causes of pineal region masses, to enable effective primary diagnosis and, consequently, correct treatment and clinical management.

### 2.1. Germ Cell Tumors

Germ cell neoplasms are derived from primordial germ cells that develop primarily in the gonads but also in the anterior mediastinum, pineal gland, and brain [31]. Germ cell neoplasms account for 0.5–3.2% of primary intracranial tumors in adults and 11.8% of the same in children. Germ cell tumors are predominantly found in male patients. Pineal germ cell tumors account for about 50% of intracranial germ cell tumors [3] and seem to be more common in Asian populations [3,32]. Germ cell tumor can be classified into six types: germinomas, choriocarcinomas, teratomas, embryonal carcinomas, yolk sac tumors and mixed germ cell tumors (characterized by the features of at least two of the above-cited tumor types) [3,13,33].

#### 2.1.1. Germinomas

Germinomas are the most common pineal tumor type, representing up to 50% of pineal tumors in Europe, the United States, and Japan [22,23]. Only 8% of central nervous system germinoma cases show the simultaneous involvement of pineal and suprasellar regions, and these are called bifocal germinomas [26,34,35]. Germinomas are not encapsulated tumors and thus may invade adjacent brain structures and, through the CSF, disseminate along the brain surface. Germinomas present cellular sheets or lobules of uniform germinoma cells with large round nuclei, prominent nucleoli, and clear cytoplasm with connective tissue septal bands and full of capillaries, lymphocytes, and, occasionally, granulomas [3,36]. Furthermore, germinomas present significant amounts of lipids and macromolecules compared to other pineal gland tumors [37,38].

Germinomas are malignant tumors characterized by a mix of large multipotential primitive germ cells and smaller cells that resemble lymphocytes. Germinomas often present severe inflammatory infiltrates [20]. Furthermore, corticosteroid treatment seems to be able to modify the patient’s immunological defense, enabling the immune system to suppress the tumor [20,39].

**Figure 3:**
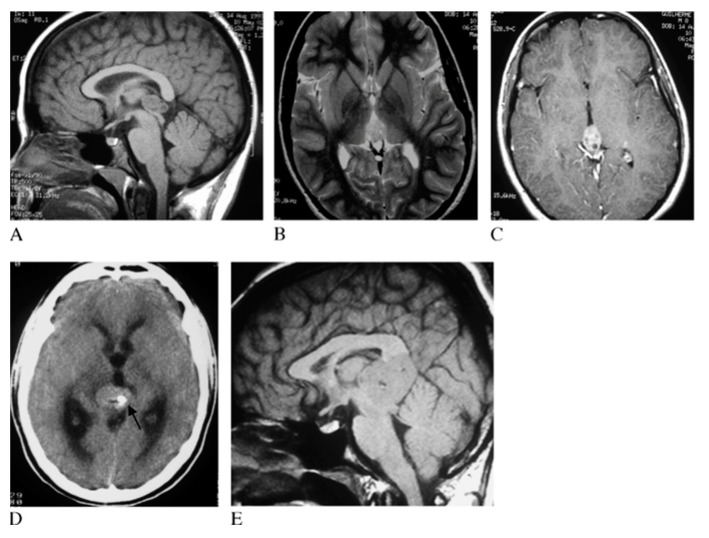
Pineal germinoma magnetic resonance imaging (MRI). Pineal tumor hypointense on T1 weighted image (T1WI) (**A**), hyperintense on T2WI (**B**) and with homogeneous contrast enhancement (**C**). The arrow identifies hyperdense mass with calcification at computed tomography (**D**). In another patient, a larger germinoma, isointense on T1WI (**E**). Reprinted with permission from Reis et al. (2006) [41]. John Wiley and Sons—2021 (License Number 5034711197310).

On imaging, germinomas show heterogeneous features, often presenting as solid or solid/cystic masses with engulfed calcifications (Figure 3), different to pineal parenchymal tumors, which exhibit prominent calcifications [14,31,40].

Imaging alone does not allow us to distinguish among germinomas, nongerminomatous germ cell tumors and pineal parenchymal tumors. Therefore, a complete evaluation is fundamental. In fact, germinomas are diagnosed using imaging together with serum and CSF markers. These tumors present high serum and CSF expression of oncoproteins such as alpha-fetoprotein, beta human chorionic gonadotropin, lactate dehydrogenase, and placental alkaline phosphatase [6].

The treatment regimens used against germinomas include chemo- and radio-therapy or a combination of both, resulting in a positive prognosis and five-year survival of at least 90% [14]. Germinomas are very radiosensitive neoplasms and respond well to specific chemotherapy [13,14,20,31,42,43,44,45]. Furthermore, for germinoma, stereotactic radiosurgery seems to be effective in improving standard adjuvant treatment or in the case of recurrence [21].

#### 2.1.2. Choriocarcinomas

**Figure 4:**
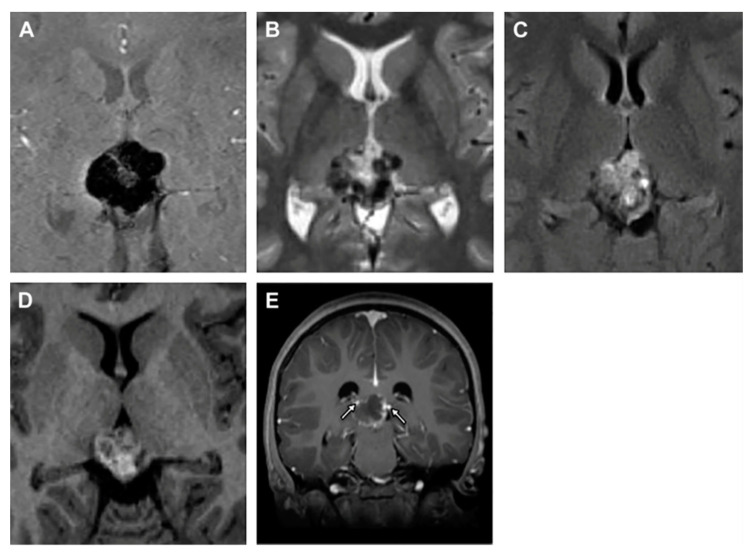
Pineal choriocarcinoma MRI. Susceptibility-weighted imaging (**A**), T2WI (**B**) and pineal heterogeneous mass on T1WI (**C**,**D**). The arrows identify the faint peripheral enhancement after the intravenous administration of gadolinium (**E**). Reprinted with permission from Causil et al. (2016) [46]. Elsevier—2021 (License Number: 5034730033295).

Pineal choriocarcinomas are uncommon malignant nongerminomatous germ cell neoplasms (accounting for fewer than 5% of all pineal masses) [13] and the most aggressive form of gestational trophoblastic disease. Choriocarcinoma shows a poor survival rate with respect to other germ cell tumors [46,47]. Median overall survival of primary intracranial pure choriocarcinoma was 22 months and the three- and five-year survival rate was 45.8% [48]. Primary intracranial choriocarcinoma mainly affects young men (3–22 years of age), who present precocious puberty. These tumors do not show distinctive symptoms, but patients affected by choriocarcinoma have mainly reported headaches, vomiting, nausea, visual impairment, polydipsia, polyuria and endocrinologic alterations [46,49]. Choriocarcinomas present stromal vascular ducts that form blood lakes and intratumoral hemorrhagic necrosis, all factors strictly correlated with a poor prognosis [2,46,50,51]. On imaging, choriocarcinomas appear as ovoid, heterogeneous, and slightly hyperdense masses (Figure 4).

Qi et al. [50] observed sinusoids that expressed laminin and not CD34, thus identifying tumor vasculogenic mimicry. Blood may flow from tumor vessels that express CD34 to sinusoids, leading to blood clotting, the extension of blood lakes and sinusoids, and, sometimes, hemorrhagic necrosis. Choriocarcinoma is also linked with elevated levels of both CSF and plasma human chorionic gonadotropin [50]. Choriocarcinomas and germinomas are both associated with elevated beta human chorionic gonadotropin expression [1,52,53].

Unfortunately, classic treatments frequently fail due to choriocarcinomas being extremely resistant tumors. The first clinical choice is total resection (even if the patient does not present hydrocephalus). However, to treat choriocarcinoma, a mix of total tumor removal, chemotherapy, and radiotherapy is frequently used as a therapeutic combination and seems to show positive outcomes [46,47,48,49].

#### 2.1.3. Teratomas

**Figure 5:**
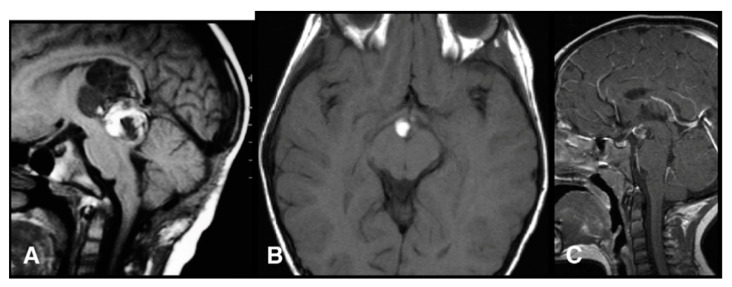
Pineal teratoma MRI. Sagittal T1 image (**A**), axial T1 (**B**) and sagittal contrast-enhanced T1 (**C**) images. Reprinted with permission from Peterson et al. (2012) [54]. Elsevier—2021 (License Number: 5034851378505).

Intracranial teratomas account for up to 50% of fetal brain neoplasms; in neonates, they comprise 33% of intracranial tumors, but they comprise only 2%–4% of intracranial tumors in patients aged <15 years [54,55]. Intracranial teratomas typically arise from the pineal gland and involve the third ventricle [54]. Pineal teratomas have a male predominance that varies from 2:1 to 8:1 and an overall survival of 90–100% [26]. Histologically, teratomas are classified into: (1) mature tumors, which show completely differentiated tissue; (2) immature tumors, which present a combination of fetal- and mature-type tissue elements and elements from all three germ layers and (3) teratomas with malignant transformation, which involves the malignant degeneration of mature tissue [14,56]. Teratomas are neoplasms characterized by multipotential cells that revert to normal organogenesis, usually producing tissues representing a combination of two or more of the embryological layers of ectoderm, mesoderm, and endoderm [13,14]. Pineal teratomas can be partially or totally encapsulated, but can also be unencapsulated and locally invasive [13]. On imaging, these pineal tumors present foci of fat, calcification and cystic regions [13,54]. On MRI, teratomas appear as lobular, multiloculated, and heterogeneously wide masses [14] (Figure 5).

### 2.2. Pineal Parenchymal Tumors

Pineal parenchymal tumors are neuroepithelial neoplasms arising from pineocytes. These tumors are uncommon accounting for fewer than 1% of all primitive central nervous system tumors and constituting 15% to 30% of pineal gland tumors [57,58]. Pineal parenchymal tumors present different features, grades, and levels of aggressiveness [33]. The World Health Organization (WHO) recognizes pineal parenchymal tumors in four distinct categories: pineocytomas, pineoblastomas, papillary pineal tumors, and pineal parenchymal tumors of intermediate differentiation [14,57,59,60].

Pineal parenchymal tumors seem to have no sexual predominance and occur most frequently in pediatric patients [13,14,58]. Patients mainly report headaches, vomiting (correlated with increased intracranial pressure due to the blockade of the ventricular system) and gait ataxia [1,26,57,61,62]. Pineal parenchymal cell tumors consistently produce a hypomelatoninemic or hypermelatoninemic state [63]. However, exogenous melatonin supplementation after pinealectomy may mitigate the ensuing syndrome [1,64,65]. Pineal parenchymal tumors are negative for the three tumor markers alpha-fetoprotein, beta human chorionic gonadotropin, and placental alkaline phosphatase [1]. However, synaptophysin is expressed in pineal parenchymal tumors of intermediate differentiation, and neuronal marker positivity is not related to histological grade, mitosis, proliferation index, or prognosis [66]. The standard treatment for pineal parenchymal tumors is radiation. Surgery is another possible treatment option; however, it has a mortality rate of 5%–10% [1,24], and even after complete tumor removal, many patients present recurrence. Hence, adjuvant radiation or chemotherapy or a mix of both is often suggested with the aim of improving survival [1,61,62]. Thus, appropriate and close follow-up is also fundamental.

#### 2.2.1. Pineocytomas

Pineocytomas are slow-growing grade I/II pineal parenchymal neoplasms derived from the pineal epithelium [1,15,51,60,67]. They are tumors characterized by well-differentiated mature cells arranged in sheets that are virtually indiscernible from the healthy pineal parenchyma [13,14,68]. They are circumscribed, unencapsulated tumors that may remain locally confined. Pineocytomas may arise at all ages but are more frequent in adults aged 30 to 60 years old [14,69].

**Figure 6:**
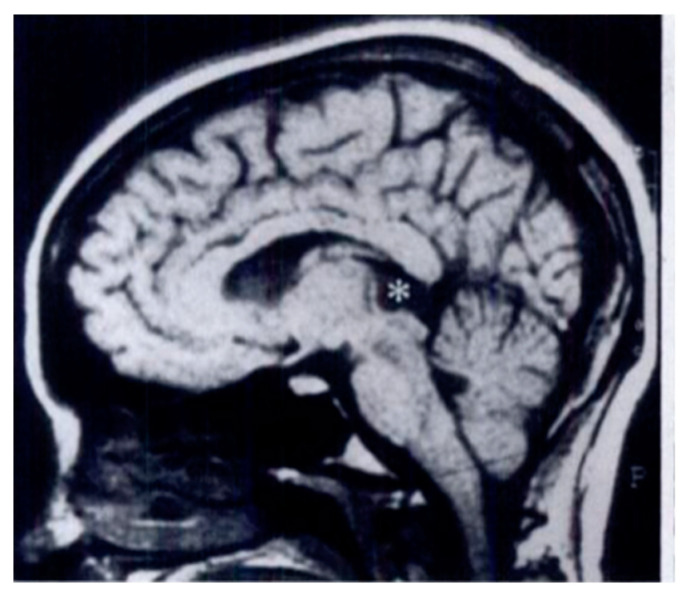
Pineocytoma MRI. The asterisk indicates the homogeneous mass in the pineal gland. Reprinted with permission from Smirniotopoulos et al. (1992) [13]. Radiological Society North America–2021. Small pineocytomas often do not induce symptoms, but if they are of large dimensions, they may induce obstructive hydrocephalus and Parinaud syndrome, defined as upward gaze palsy, pupillary light-near dissociation, and convergence retraction nystagmus [15]. The five- and twenty-year survival rates are, respectively, 100% and 76% [21,26]. For pineocytomas, stereotactic radiosurgery seems to be highly effective as a primary treatment, so stereotactic radiosurgery alone may be considered appropriate clinical management for pineocytomas [21,28,71,72].

On MRI, pineocytomas appear as hyperintense, round or lobular masses [2,14,15,56,70] (Figure 6).

#### 2.2.2. Pineoblastomas

**Figure 7:**
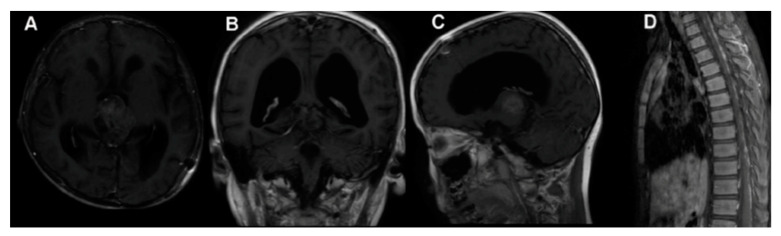
Pineoblastoma MRI. Preoperative MRI (**A**–**C**), MRI 1.5 months after surgery showing spinal metastasis (**D**). Reprinted with permission from Huo et al. (2020) [79]. Dove Medical Press—2021.

As previously reported, pineal parenchymal cell tumors also include pineoblastomas, aggressive, grade IV neoplasms derived from primitive neuroectoderm [1,17]. They account for 40% of parenchymal pineal cancers. Pineoblastomas are undifferentiated, embryonal tumors categorized as primitive neuroectodermal tumors. Similar to other primitive neuroectodermal tumors, pineoblastomas are extremely aggressive and present a poor prognosis and aggressive clinical behavior due to the frequent invasion of adjacent structure and CSF dissemination, with reported five-year survival rates of <60% [23,57,73,74]. The highest incidence is in childhood, particularly in children less than 2 years old, where pineoblastomas can occur in combination with retinoblastomas [13,14,26,59,75]. Adult cases are very rare, and for this reason, limited data are currently available for the effort to develop a standard management course for the diseases. Pineoblastomas are malignant, unencapsulated tumors that, unfortunately, may often recur and also disseminate throughout the craniospinal axis, as well as presenting metastasis all over the body, such as in the calvarial bones, vertebrae, lungs, peritoneum, mandible, and pelvis [59,76,77,78] (Figure 7).

Pineoblastomas are composed of undifferentiated or immature pineal cells [13,74]. Synaptophysin and chromogranin are markers of primitive neuroendocrine tumors that may be expressed in pineoblastomas and detectable in the serum or CSF [1,80]. The molecular background of pineoblastoma is not clearly known. However, recent studies showed that the DICER1 and DROSHA genes, involved in microRNA dysregulation, are fundamental to pineoblastoma carcinogenesis [81,82].

Aggressive surgical resection is the first-line therapy against pineoblastoma. Notably, radiotherapy after resection helps in increasing patient survival. As also reported for other pineal gland tumors, combined therapy seems to be an effective approach [59]. A report from the International Gamma Knife Research Foundation described actuarial local control and survival rates following the stereotactic radiosurgery of pineoblastomas of 27% and 48% at 5 years [29]. Recently, Jing et al. [59] evaluated 213 adult patients with pineoblastomas and observed that young patients, especially those not receiving radiotherapy during their initial treatment, often had extremely poor outcomes [59,83]. This study suggested that beam radiotherapy does not improve overall patient survival. Furthermore, tumors that invade tissue near the pineal gland are more aggressive than tumors confined to the pineal gland, increasing the risk of patient death about threefold. Jing et al. [59] also reported that, for every 1 mm increase in tumor size, the risk of death is reduced by 4%, so tumor size may not be considered a prognostic factor.

#### 2.2.3. Papillary Tumors

Pineal papillary tumors are uncommon WHO grade II or III neuroepithelial neoplasms [14,15,56]. Among them, 68% have been found to recur at a mean follow-up of 4.2 years (with overall survival rates of 73% at 5 years and 58% at 10 years), and in a pediatric population, 47% of papillary tumors of the pineal gland recurred at a mean follow-up of 6.5 years [83,84]. The age of patients presenting pineal papillary tumors is 15 months to 67 years and there is a mild female prevalence. The main symptom reported by patients with pineal papillary tumors is headaches, which are related to obstructive hydrocephalus [85,86,87,88,89].

**Figure 8:**
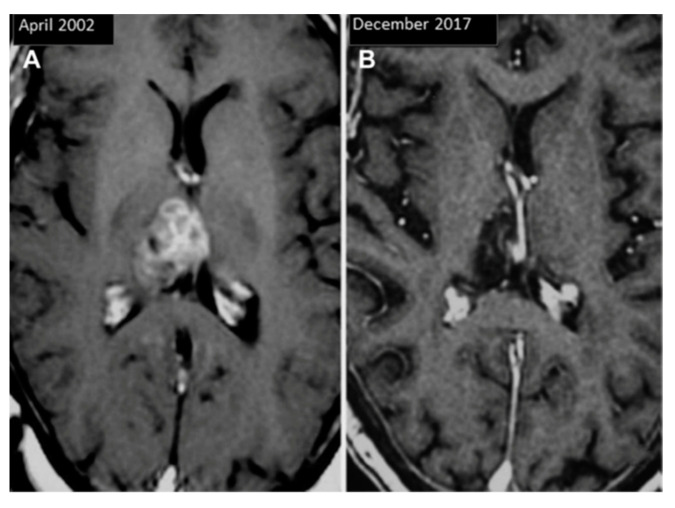
Pineal papillary tumor MRI before (**A**) and after 15 years of treatments (**B**). Reprinted with permission from Fernández-Mateos et al. (2018) [90]. Elsevier—2021 (License Number: 5034870401490).

Pineal papillary tumors at MRI appear as partially cystic masses with obstructive hydrocephalus (Figure 8).

Pineal papillary tumors show some papillary morphological features forming ependymal rosettes and pseudorosettes. The rosetted cells have thick processes resting on collagen from adjacent blood vessels. Vascular connective tissue present various layers of cells with a large cuboidal or columnar epithelial-like growth pattern. These cells show small round/oval nuclei, stippled chromatin, small nucleoli, and eosinophilic cytoplasm with distinct cell borders [61,83,85,90,91]. Mitotic figures are uncommon, whereas necrosis is often present [83,91]. It is important to underline that pineal papillary tumors present wide morphological variability [82,92,93,94,95]. Apart from the characteristic papillary structures, papillary tumors have morphological features in common with other papillary-like tumors that occur in the pineal region, including pineal parenchymal neoplasms, choroid plexus papillomas, papillary ependymomas, metastatic papillary carcinomas, papillary meningiomas, and germ cell tumors, which complicates the clinical diagnosis [60,83,87,92,96,97]. These pineal tumors often present increased proliferative activity (Ki67/MIB1 proliferation index) [90], which is correlated with worse prognosis. Immunohistochemically, papillary tumors are also positive for S100, CAM 5.2, and prealbumin. Pineal papillary tumors usually show neuron-specific enolase reactivity but do not present neurofilament proteins—interesting papillary tumor features that may help in differentiating them from pineal parenchymal tumors with intermediate differentiation, whereas—MAP-2 is used for differentiating them from choroid plexus papillomas in diagnosis [17,60,98,99].

Pineal papillary tumors often recur, and radiotherapy is frequently effective [14,60,92,99,100]. Radiotherapy and chemotherapy are both used in initial tumor management and in cases of recurrences after gross total resection; however, the optimal adjuvant therapy is actually not known [99]. Stereotactic radiosurgery seems to be effective against pineal papillary tumors, but a high rate of local recurrence is also observed after this treatment. Recurrence can be safely and successfully managed by repeat stereotactic radiosurgery [21].

#### 2.2.4. Pineal Parenchymal Tumors of Intermediate Differentiation

**Figure 9:**
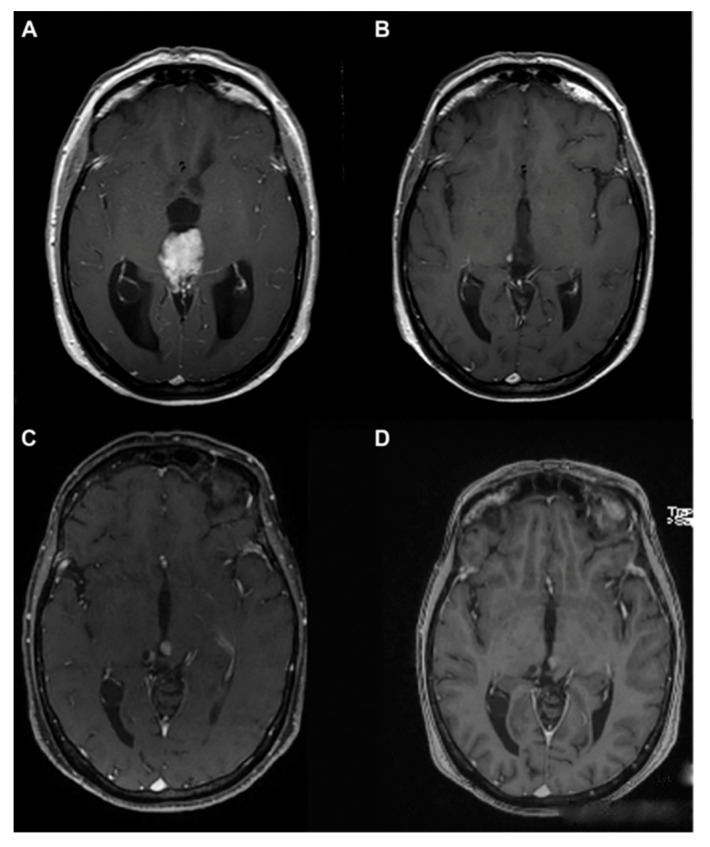
Pineal parenchymal tumors of intermediate differentiation MRI. Preoperative MRI (**A**), postoperative MRI showing a very small residual pineal lesion (**B**). MRI before (**C**) and after (**D**) radiation therapy. Reprinted with permission from Choque- Velasquez et al. (2019) [102]. Elsevier—2021 (License Number: 5035440109275). Pineal parenchymal tumors of intermediate differentiation may be morphologically classified into (1) lobulated endocrine-like and highly vascular lesions; (2) those with diffuse growth patterns, similar to oligodendrogliomaneurocytomas; and (3) those of transitional type, with areas of lobulated and diffuse growth patterns, correlated with areas of pineocytomatous rosettes [82,102,109]. Recently, Wu et al. [103] reported that CD24 and PRAME may be novel markers useful for the grading and prognostic evaluation of pineal parenchymal tumors of intermediate differentiation and, thus, useful in therapeutic decision-making. The best treatment for pineal parenchymal tumors of intermediate differentiation has not yet been found, partly due to the low numbers of reported cases. The maximal surgical removal of the tumor is the optimal treatment in practice for pineal parenchymal tumors of intermediate differentiation. However, even after complete surgical tumor removal, many patients experience recurrence. Hence, adjuvant radio- or chemo-therapy, or a mix of both, is often recommended to improve patient survival. A report from the International Gamma Knife Research Foundation described actuarial local control and survival rates following stereotactic radiosurgery on pineal parenchymal tumors of intermediate differentiation of 50% and 56%, respectively, at 5 years [19,26].

Pineal parenchymal tumors of intermediate differentiation are uncommon tumors arising from the pineal parenchyma that present features between those of pineocytomas and pineoblastomas [62,101,102,103]. Pineal parenchymal tumors of intermediate differentiation can occur at all ages. They appear more commonly among women, teenagers, and middle-aged patients [51,58,62,101]. Pineal parenchymal tumors of intermediate differentiation are often considered to be not a single disease, but a spectrum of grade II and III pineal parenchymal tumors, thus explaining the huge variation in the behavior of these tumors [101,104]. Although grading criteria for differentiating pineal parenchymal tumors of intermediate differentiation of grades II and III have not actually been established, the 2007 WHO Classification of Central Nervous System Tumors considers as outcome predictors both proliferative activity and immunoreactivity for neurofilament protein [102,105]. Thus, WHO grade II pineal parenchymal tumors of intermediate differentiation may present as lobulated or diffuse with a higher expression of neurofilaments, 0–5 mitoses per 10 high-power fields and moderate MIB1 labeling indices (ranging from 3% to 10% [101,106,107,108]). Pineal parenchymal tumors of intermediate differentiation show moderate cellularity, mild-to-moderate atypical nuclei, and low-to-moderate mitoses [101]. Choque-Velasquez et al. [102] reported that, on MRI, pineal parenchymal tumors of intermediate differentiation tend to appear as isointense lesions on T1WI and hypointense masses on T2WI, with some cystic components, and a complete contrast enhancement (Figure 9). Multiple cystic elements may also be observed. Pineal calcifications may be engulfed or peripherally displaced by the mass [15]. These tumors present positive expression for synaptophysin, neurofilament, chromogranin A, and renal S antigen.

## 3. A Brief Overview of Other Neoplastic and Non-Neoplastic Pineal Masses

### 3.1. Pineal Metastasis

**Figure 10:**
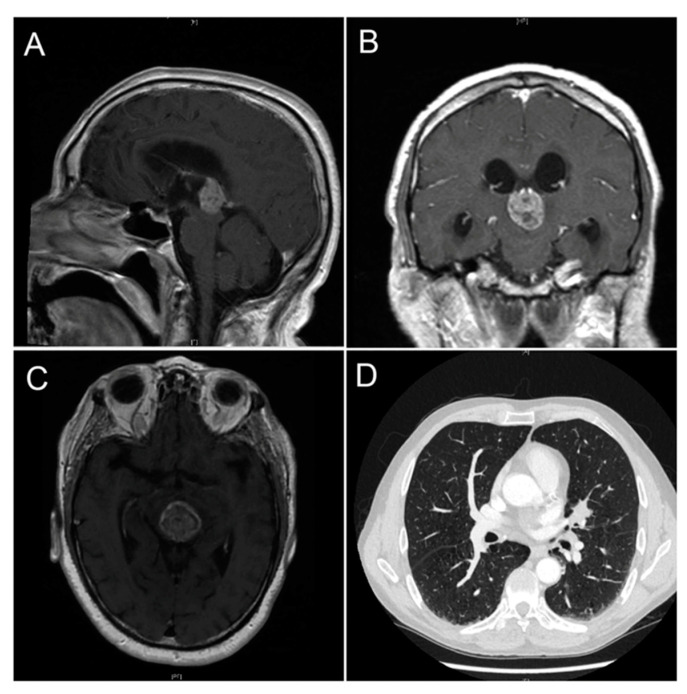
Pineal metastasis from lung adenocarcinoma. Brain MRI sagittal (**A**), coronal (**B**) and transverse (**C**) sections showing the pineal gland metastatic mass. Computed tomography showing a lung nodule over the left hilar region in the lower right corner (**D**). Image from Abdallah et al. (2020) [110]—Ochsner Journal.

Metastatic cancer spreading to the pineal gland is exceedingly uncommon and originates from lung carcinoma in most cases (Figure 10). Other tumors that have documented cases of metastases to the pineal gland are pancreatic, esophageal and bladder neoplasms [110,111,112,113].

Metastasis to the central nervous system is usually an end-stage disease features and the treatment of pineal region metastases varies due to systemic conditions and neurological symptoms [111,112]. However, Hogan et al. [111] described, for the first time, a successful treatment of metastatic prostate cancer that had spread to the pineal gland. The pineal metastasis was treated with stereotactic radiosurgery, and, after 9 months, the pineal metastatic mass was significantly smaller.

Blas John et al. [114] discussed an uncommon case of an adult woman with an esophageal primitive neuroectodermal tumor that presented pineal gland metastasis. The head computed tomography of the patient showed tumor invasion of the third ventricle, with incipient hydrocephalus. The patient underwent a craniotomy of the posterior fossa with a supracerebellar infratentorial approach to the pineal region and an external ventricle drainage. Histopathologically, the tumor appeared rich in cellular density, forming lobes. The metastatic cells showed scant cytoplasm, elongated atypical nuclei, and abundant mitotic figures with up to 10 mitoses in one high-power field. Foci of necrosis and areas with abundant electrocautery artifacts were also observed. These areas had a lower cellular density and peripheral calcification, suggestive of a pineoblastoma. In terms of imunohistochemistry the pineal tumor showed a patchy expression of synaptophysin, a diffuse and strong expression of CD99 and weak expression of the cytokeratins AE1/AE3 and CAM 5.2. Partial resection of the pineal metastasis was performed. The patient was given chemotherapy; however, tumor progression was observed.

### 3.2. Pineal Cysts

Non-neoplastic pineal cysts are frequently observed during both MRI scans and autopsy studies, and the prevalence of benign pineal cysts ranges between 0.6% and 23% in the general population [115,116,117,118,119,120,121]. Pineal cysts, usually considered normal anatomical variations, are present at all ages but mainly in adult women in the fourth decade of life. Symptomatic cysts are more frequent among young women [116,119,122].

**Figure 11:**
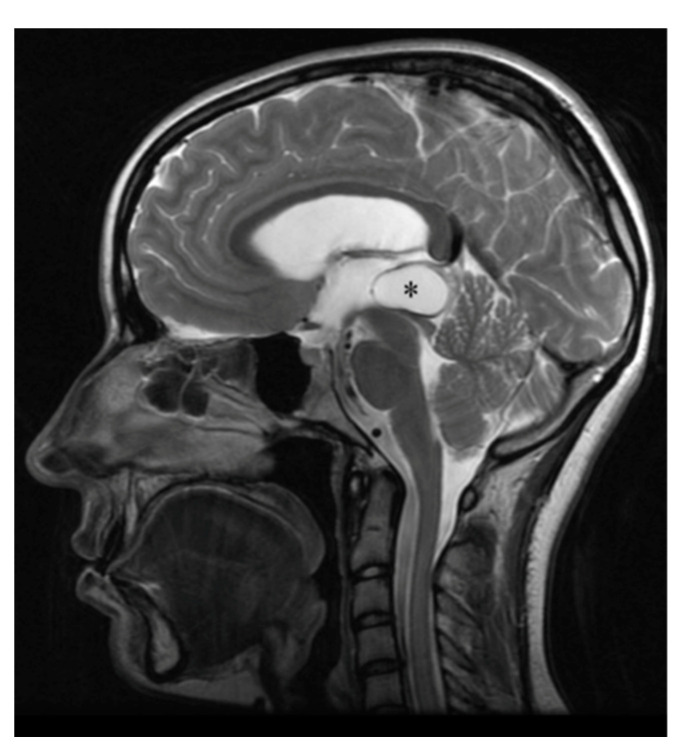
Pineal cyst MRI. The asterisk indicates the pineal cyst. Reprinted with permission from Májovský et al. (2017) [124]—Elsevier—2021 (License Number 5020240575766).

It is, in our opinion, important to at least introduce pineal cysts because they are pineal non-neoplastic masses that are relatively common in the general population, and a heterogeneous group of tumors, such as pineoblastomas, astrocytomas, meningiomas and pineocytomas, may feature a cystic nature on MRI, without showing hemorrhage within the masses [119,123]. Pineal cysts present an inner glial layer, a middle layer of pineal tissue and an outer fibrous capsule. On MRI scans, benign pineal cysts appear as round or ovoid areas of signal abnormality in the pineal region [120,124] (Figure 11).

Barboriak et al. [125] undertook a follow-up MRI study and, interestingly, observed that pineal cysts usually remain stable. Only a few pineal cyst cases may enlarge, making them of neurological significance. Large cysts typically exert a mass effect on the cerebral aqueduct, encircling venous structures and the dorsal midbrain. The most reported symptoms, correlated to the compression of the surrounding structures, are headaches, vertigo, visual and oculomotor disturbances, and obstructive hydrocephalus [117,119,125]. Symptomatic pineal cysts are divided into three syndromes: (1) paroxysmal headaches and gaze palsy; (2) chronic headaches, papilledema, gaze paresis, and hydrocephalus; and (3) pineal apoplexy with acute hydrocephalus. The last is the least frequent but most dangerous form. In practice, there are no accepted therapeutic indications or criteria for intervention and/or follow-up. However, only a few patients with pineal cysts requires treatment. In fact, as previously reported, pineal cysts usually have no clinical implications and are asymptomatic [119,126,127].

Choque-Velasquez et al. [117] reported in their recent case series that surgically treated pineal cysts represent a progressive disease with acute or progressive hydrocephalus at the final stage. The authors also suggested that young women with active sexual hormone status (aged >10-years) comprise the patient group at the highest risk for pineal cyst progression.

## 4. Conclusions

As reported in the present review and summarized in Table 1, pineal neoplasms are heterogeneous tumors with different histological, morphological, and radiological features and, consequently, different diagnosis and management.

Furthermore, due to their rarity, pineal tumors may be misdiagnosed. Establishing a means of specific tissue diagnosis for patients with pineal region tumors is needed to distinguish among the variety of possible histomorphological tumor subtypes. In the last decade, specialized surgical and stereotactic techniques have evolved to provide specific, safer, and more effective options for obtaining, at least, correct tissue diagnosis. Advanced microsurgical techniques combined with improved preoperative management and postoperative critical care methods have made surgical resection the optimal therapy for almost all pineal tumors. However, the identification of new therapeutic targets and the development of more effective adjuvant therapies, as well as follow-up guidelines, are needed to improve pineal tumor outcomes.

## Figures and Tables

**Table 1:** The table summarizes the main characteristics of pineal gland tumors.

	Histological Subtype	Morphology/Histology	Incidence, Age and Sex Distribution
Germ Cell Tumors	Germinomas	Not encapsulated tumors with variable proportions of germinoma cellular sheets or lobules composed of a mixture of large multipotential primitive germ cells and smaller cells that resemble lymphocytes. Often presented inflammatory infiltrates.	Most common pineal tumor (>50% of pineal tumors in Europe, the United States, and Japan). Male predominance.
	Choriocarcinomas	Tumors with stromal vascular channels that form blood lakes and intratumoral hemorrhagic necrosis.	Rare pineal tumor (<5% of all pineal masses). Young men predominance (3–22 years of age).
	Teratomas	Encapsulated tumors with multipotential cells that recapitulate normal organogenesis. (Teratoma may also be unencapsulated).	Second most common pineal tumors. Male predominance.
Pineal Parenchymal Tumor	Pineocytomas	Unencapsulate tumors with well-differentiated mature cells arranged in sheets. Pineocytic rosettes.	About 14–30% of all pineal parenchymal tumors. More common in adults (30–60 years old).
	Pineoblastomas	Undifferentiated or immature pineal cells.	40% of all pineal parenchymal tumors. Highest incidence in children less of 2 years old. Slightly female predominance.
	Papillary Tumors	Tumors with partially papillary structures lined by slightly polymorphic cells forming ependymal rosettes and pseudorosettes. The rosetted cells had thick processes resting on collagen surrounding the vessels.	Rare pineal parenchymal tumors.
	Pineal Parenchymal Tumors of Intermediate Differentiation	Pseudolobulated architecture with multiple cystic components.	Rare pineal parenchymal tumors which may occur in all ages. Slightly female predominance (teenagers and middle-aged women).
